# mTORC1 Signaling Is Palmitoylation-Dependent in Hippocampal Neurons and Non-neuronal Cells and Involves Dynamic Palmitoylation of LAMTOR1 and mTOR

**DOI:** 10.3389/fncel.2019.00115

**Published:** 2019-04-02

**Authors:** Shaun S. Sanders, Francesca I. De Simone, Gareth M. Thomas

**Affiliations:** ^1^Shriners Hospitals Pediatric Research Center, Lewis Katz School of Medicine at Temple University, Philadelphia, PA, United States; ^2^Department of Anatomy and Cell Biology, Lewis Katz School of Medicine at Temple University, Philadelphia, PA, United States

**Keywords:** mTOR, mTORC1, LAMTOR1, palmitoylation, neurotrophins, BDNF

## Abstract

The mechanistic target of rapamycin (mTOR) Complex 1 (mTORC1) controls growth and proliferation of non-neuronal cells, while during neuronal development mTORC1 responds to glutamate and neurotrophins to promote neuronal migration and dendritic arborization. Recent studies reveal that mTORC1 signaling complexes are assembled on lysosomal membranes, but how mTORC1 membrane targeting is regulated is not fully clear. Our examination of palmitoyl-proteomic databases and additional bioinformatic analyses revealed that several mTORC1 proteins are predicted to undergo covalent modification with the lipid palmitate. This process, palmitoylation, can dynamically target proteins to specific membranes but its roles in mTORC1 signaling are not well described. Strikingly, we found that acute pharmacological inhibition of palmitoylation prevents amino acid-dependent mTORC1 activation in HEK293T cells and brain-derived neurotrophic factor (BDNF)-dependent mTORC1 activation in hippocampal neurons. We sought to define the molecular basis for this finding and found that the mTORC1 proteins LAMTOR1 and mTOR itself are directly palmitoylated, while several other mTORC1 proteins are not palmitoylated, despite strong bioinformatic prediction. Interestingly, palmitoylation of LAMTOR1, whose anchoring on lysosomal membranes is important for mTORC1 signaling, was rapidly increased prior to mTORC1 activation. In contrast, mTOR palmitoylation was decreased by stimuli that activate mTORC1. These findings reveal that specific key components of the mTOR pathway are dynamically palmitoylated, suggesting that palmitoylation is not merely permissive for mTOR activation but is instead actively involved in mTORC1-dependent signaling.

## Introduction

Mechanistic (previously mammalian) target of rapamycin (mTOR) signaling plays a key role in many processes that are essential for normal cell growth and survival, including protein synthesis, transcription, cytoskeletal regulation, autophagy, and neuronal morphology. Conversely, mTOR signaling is overactivated in many disease states including cancer, type 2 diabetes, and a group of neurodevelopmental disorders termed “mTORopathies,” which are characterized by epilepsy and/or Intellectual Disability (Laplante and Sabatini, 2012; Costa-Mattioli and Monteggia, 2013; Crino, 2016).

mTOR is a protein serine/threonine kinase that belongs to the phosphatidylinositol 3-kinase-related kinase family and is the catalytic subunit for two distinct protein complexes. These two complexes termed mTOR Complex 1 (mTORC1) and 2 (mTORC2) have unique protein components and respond to different cellular signaling events with distinct outcomes (Laplante and Sabatini, 2009). mTORC1 is activated by diverse intracellular and extracellular cues, including growth factors, stress, energy status, oxygen, and amino acids (AA), to promote protein and lipid synthesis and inhibit autophagy (Laplante and Sabatini, 2012; Ben-Sahra and Manning, 2017). mTORC1 is thought to be activated by at least two mechanisms. Growth factors and insulin stimulate Phosphatidylinositide 3-kinase-Akt signaling to phosphorylate and relieve inhibition by the negative mTORC1 regulator tuberous sclerosis complex 2 (TSC2), allowing mTORC1 activation by the small GTPase Ras highly expressed in brain (Rheb; Ben-Sahra and Manning, 2017). A similar mechanism is also implicated in neuronal mTORC1 activation by the neurotrophin brain-derived neurotrophic factor (BDNF; Takei et al., 2004). mTORC1 is also activated by a second mechanism that involves not just Rheb but also the Ras-related GTP-binding (Rag) family of GTPases, which are activated following exposure of cells to nutrients, such as amino acids (Costa-Mattioli and Monteggia, 2013).

Interestingly, a key step in both of these mTORC1 activation mechanisms is the recruitment of mTOR to the surface of lysosomes (Sancak et al., 2008, 2010; Efeyan et al., 2012; Betz and Hall, 2013; Averous et al., 2014). Moreover, several other mTORC1 regulators are also either constitutively present on lysosomal membranes, or translocate to or from lysosomes during mTOR signaling (Sancak et al., 2010; Menon et al., 2014). However, the mechanisms underlying these membrane localization and translocation events are not fully understood (Betz and Hall, 2013; Averous et al., 2014).

**Figure 1:**
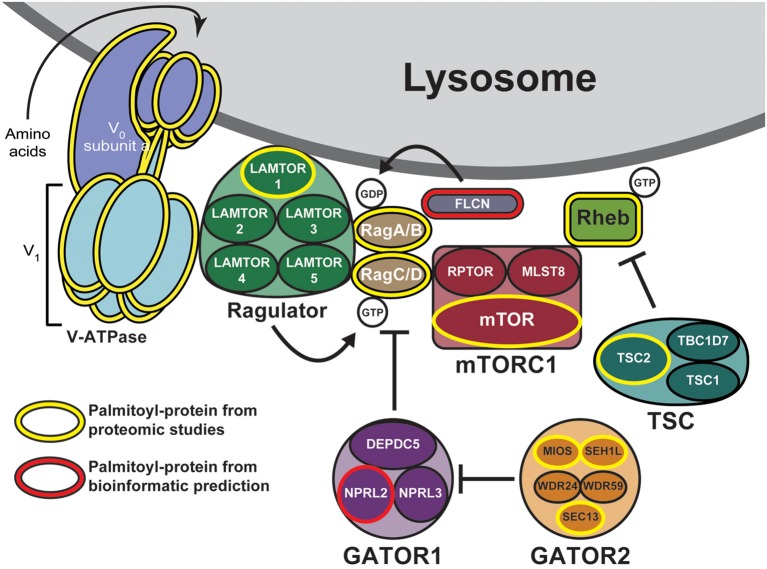
Putative palmitoylated proteins of the mechanistic target of rapamycin (mTOR) signaling pathway. Palmitoyl-proteomic studies have identified a number of mTOR signaling components as potentially palmitoylated (outlined in yellow) including mTOR itself, subunits of the vacuolar ATPase, components of the regulatory GATOR2, Ragulator, and tuberous sclerosis complex (TSC) complexes, and other regulatory proteins. Additional mTOR signaling components are bioinformatically predicted to be palmitoylated (outlined in red).

One mechanism to dynamically control protein localization in response to signals is palmitoylation. This process, also known as S-acylation, is the posttranslational addition of long-chain fatty acids, typically palmitate, to protein cysteine residues, and often targets proteins to specific membranes and/or membrane subdomains (Hallak et al., 1994; Smotrys and Linder, 2004). Palmitoylation is the only reversible protein-lipid modification and is thus mechanistically analogous to phosphorylation. However, instead of a charged phosphate group being added and removed by kinases and phosphatases, respectively, the hydrophobic palmitate lipid is added by palmitoyl acyltransferases and removed by acyl protein thioesterases (Zeidman et al., 2009; Tomatis et al., 2010). Proteins can thus undergo cycles of palmitoylation and depalmitoylation in response to extracellular and intracellular signaling events, thereby leading to dynamic changes in protein localization and/or function (El-Husseini et al., 2002; Martin et al., 2012; Brigidi et al., 2015). Strikingly, recent curations of multiple palmitoyl-proteomic studies revealed that numerous mTOR pathway components are potentially palmitoylated (Table 1 and Figure 1; Blanc et al., 2015; Sanders et al., 2015). However, the functional importance of palmitoylation for mTOR localization and activation has not been addressed.

**Table 1:** Mechanistic target of rapamycin (mTORC1) signaling proteins in palmitoyl-proteomic databases.

Complex	Protein	# of studies^a^	# of techniques^b^	Validation
GATOR2	SEC13	2	2	–
	SEH1	2	2	–
	WDR24	0	–	–
	WDR59	0	–	–
	MIOS	1^c^	1	–
GATOR1	DEPD5	0	–	–
	NPRL2	0	–	–
	NPRL3	0	–	–
mTORC1	mTOR	1^c^	1	–
	LST8	0	–	–
	RPTOR	0	–	–
	DPTOR	0	–	–
Ragulator	LAMTOR1	17	2	Martin and Cravatt (2009)
	LAMTOR2	0	–	–
	LAMTOR3	0	–	–
	LAMTOR4	0	–	–
	LAMTOR5	0	–	–
–	RagA	1	1	–
	RagB	0	–	–
	RagC	0	–	–
	RagD	1	1	–
V-ATPase	ATP6V0A1	3	2	Bagh et al. (2017)
	ATP6V0A2	7	2	–
	ATP6V0A3	0	–	–
	ATP6V0A4	0	–	–
	ATP6V0B	0	–	–
	ATP6V0C	1	1	–
	ATP6V0D1	3	2	–
	ATP6V0D2	0	–	–
	ATP6V0E1	0	–	–
	ATP6V0E2	0	–	–
	ATP6V1A1	10	2	–
	ATP6V1B1	0	–	–
	ATP6V1B2	5	2	–
	ATP6V1C1	3	2	–
	ATP6V1C2	0	–	–
	ATP6V1D	1	1	False positive, no Cys
	ATP6V1E1	2	2	–
	ATP6V1E2	0	–	–
	ATP6V1F	0	–	–
	ATP6V1G1	0	–	–
	ATP6V1G2	0	–	–
	ATP6V1G3	0	–	–
	ATP6V1H	5	2	–
TSC	TSC1	0	–	–
	TSC2	1	1	–
	TBC1D7	0	–	–
FLCN	FLCN	0	–	–
RHEB	RHEB	1	1	–

*^a^Human, mouse, rat studies manually curated from SwissPalm (Blanc et al., 2015) unless otherwise indicated (31 total as of 10/2018). ^b^Number of techniques: ABE-based (including Acyl-RAC) and/or bioorthogonal labeling. ^c^Identified in mammalian studies from Sanders et al. (2015)*.

Here, we report that activation of mTORC1 by amino acids in HEK293T cells and by BDNF in hippocampal neurons is prevented by acute treatment with a palmitoylation inhibitor, 2-bromopalmitate (2BP). Mechanistically, LAMTOR1, a key component of the Ragulator complex that is required for lysosomal targeting of mTORC1, and mTOR itself are both palmitoylated, while a number of other mTORC1 pathway proteins are not. Interestingly, following amino acid activation of mTORC1 in HEK293T cells, palmitoylation of LAMTOR1 increases rapidly but transiently, while mTOR palmitoylation slowly decreases. In neurons, palmitoyl-site mutation dramatically shifts LAMTOR1 subcellular distribution from lysosomal to endoplasmic reticulum (ER) membranes. However, in contrast to its key role in activating mTORC1 in non-neuronal cells, LAMTOR1 (and hence its palmitoylation) is dispensable for BDNF-induced mTORC1 activation in neurons. While the key palmitoyl-protein(s) that control mTORC1 activity thus remains to be identified, these findings reveal a novel palmitoylation-dependence of mTORC1 activation that allows for dynamic control of cellular responses to a variety of stimuli.

## Materials and Methods

### Antibodies

The following antibodies were used for Western blotting: phospho-S6 Ser240/244 [Cat# 2215, 1:1,000, Cell Signaling Technology (CST), Danvers, MA, USA], S6 total (#2317, 1:1,000, CST), phospho-p70 S6K-T389 (#9206, 1:1,000, CST), total p70 S6K (#2708, 1:1,000, CST), tubulin (#T7451, 1:100,000, Millipore-Sigma, St. Louis, MO, USA), Myc 9E10 (#3207, 1:250, University of Pennsylvania Cell Center), HA.11 ascites (#901514, 1:5,000, Biolegend, San Diego, CA, USA), GFP (#A-11122, 1:1,000, Thermo Fisher Scientific, Waltham, MA, USA), mTOR (#2983, 1:1,000, CST), LAMTOR1 (#8975, 1:1,000, CST), LAMTOR2 (#8145, 1:1000, CST), LAMTOR3 (#8168, 1:1,000, CST), GAP43 (#NB300-143, 1:5,000, Novus Biologicals, Centennial, CO, USA), IR-Dye 800 CW goat anti-rabbit (#926-68071, 1:10,000, LI-COR Biosciences, Lincoln, NE, USA), IR-Dye 680RD goat anti-mouse (#926-68180, 1:10,000, LI-COR Biosciences, Lincoln, NE, USA), sheep anti-mouse horse radish peroxidase (HRP)-linked whole antibody (#NA931, Millipore-Sigma), and donkey anti-rabbit HRP-linked (#711-0350152, 1:5,000, Jackson Immunoresearch Laboratories, Inc., West Grove, PA, USA). The following antibodies were used for immunostaining: Myc (#2278, 1:500, CST), GFP (#AB16901, 1:500, Millipore-Sigma, Burlington, MA, USA), GM130 (#610822, 1:500, BD Biosciences, San Jose, CA, USA), EEA1 (#610457, 1:200, BD Biosciences, San Jose, CA, USA), Ds-Red (#632496, 1:500, Clontech, CA, USA), AlexaFluor 488 goat anti-chicken (#A-11039, Thermo Fisher Scientific, Waltham, MA, USA), and AlexaFluor 647 goat anti-rabbit (#A-21244, Thermo Fisher Scientific, Waltham, MA, USA).

### HEK293T and HeLa Cell Culture and Amino Acid Stimulations

HEK293T and HeLa cells were cultured in Dulbecco’s Modified Eagle Medium (DMEM, Thermo Fisher Scientific) with 10% fetal bovine serum (FBS), 1% Penicillin-Streptomycin (Thermo Fisher Scientific), and 1× GlutaMAX Supplement (Thermo Fisher Scientific). For amino acid stimulations cells were plated on Poly-L-Lysine (Millipore-Sigma) coated plates. 24 h later cells were placed in serum-free DMEM for 16 h and then incubated in Earle’s Balanced Salt Solution (EBSS: pH 7.4, 1.8 mM CaCl_2_, 5.3 mM KCl, 0.8 mM MgSO_4_–7H_2_O, 117 mM NaCl, 26 mM NaHCO_3_, 1 μM NaH_2_PO_4_, 1% glucose). Two hours later cells were stimulated with 2× MEM non-essential amino acids (Thermo Fisher Scientific) for 30 min unless otherwise indicated. Fifteen minutes prior to stimulation cells were treated with 20 μM 2BP (Millipore-Sigma, stock solution was 20 mM in 100% ethanol) or ethanol. Cells were lysed in immunoprecipitation buffer [IPB: 1× phosphate buffered saline (PBS), 1% (w/v) Triton X-100, 50 mM NaF, 5 mM Na_4_P_2_O_7_, 1 mM Na_3_VO_4_, 1 mM EDTA, and 1 mM EGTA plus protease inhibitor cocktail (PIC, Roche, Indianapolis, IN, USA) and 1 μM microcystin] with 1× SDS sample buffer [SB: 2% SDS (w/v), 50 mM Tris pH 6.8, 10% (v/v) glycerol, 0.005% (w/v) bromophenol blue, and 1% (v/v) β-mercaptoethanol] for phospho-blots or processed for Acyl Biotin Exchange (ABE) as described below.

### Cultured Hippocampal Neurons and BDNF/Leucine Stimulations

Hippocampal neurons were dissociated from dissected embryonic day 18 rat hippocampi and cultured in Neurobasal media with B27 supplement (Thermo Fisher Scientific) as previously described (Thomas et al., 2012). At 14 days *in vitro* (DIV14), medium was changed to [artificial cerebrospinal fluid (ACSF): 25 mM HEPES pH 7.4, 120 mM NaCl, 5 mM KCl, 2 mM CaCl_2_, 20 mM glucose, 1 mM MgCl_2_] for 2 h prior to the addition of 100 ng/mL of BDNF (#ab9794, Abcam, Cambridge, United Kingdom, stock solution was 100 μg/mL) or 5 mM Leucine (MP Biomedicals, Santa Ana, CA, USA). Cells were treated 15 min before BDNF/Leucine application with 50 μM 2BP (stock solution was 50 mM in ethanol) or 100 nM rapamycin (Cayman Chemical, 13346, stock solution was 100 μM in ethanol). 30 min later, cells were lysed in IPB containing 1× SDS SB or for ABE as described below. This study was carried out in accordance with NIH guidelines and the Institutional Animal Care and Use Committee of Temple University. The protocol was approved by the Institutional Animal Care and Use Committee of Temple University.

### Western Blot and Quantification

Lysates or ABE samples were run on SDS-PAGE gels and transferred to Nitrocellulose (phospho blots; #1620112, 0.2 μm, Bio-Rad Laboratories, Hercules, CA, USA) or Immobilon-P PVDF (ABE blots; #IPVH00010, 0.45 μm, Millipore-Sigma) membranes, blocked in 5% (w/v) skim milk/Tris-buffered saline (TBS) and blotted with the indicated antibody. Blots were subsequently probed with IR-Dye fluorescent secondaries for imaging on the LI-COR Odyssey Imaging System for phospho blots or HRP conjugated secondaries for ECL-mediated visualization (Western Lightening Plus-ECL, #NEL105001EA, Perkin Elmer, Waltham, MA, USA) and film (GeneMate Blue Lite Autorad Film, F-#9024-8×10, VWR, Radnor, PA, USA) for ABE blots. Image Studio Lite was used for all Western blot quantification and data were analyzed using the statistical test indicated in the figure legend using Prism 5 software. Error bars indicate standard error of the mean and in all graphs the mean is indicated. Uncropped Western blot images are shown in Supplementary Figures S4, S5.

### Molecular Biology and cDNA Clones

Mouse LAMTOR1 cDNA was gene synthesized by Genewiz (South Plainfield, NJ, USA) and subcloned into the FEW lentiviral vector with a C-terminal Myc tag (Lois et al., 2002; Holland et al., 2016). Human LAMTOR1 cDNA was from the DNASU Plasmid Repository (Temple, AZ, USA) and was subcloned into FEWmyc or FEW GFP vectors. LAMTOR1-Myc CCSS was generated by mutating cysteines 3 and 4 to serine using mutagenic primers. A *Lamtor1* shRNA (GCGTGGATGCGAAAGAAGA) was subcloned into a modified FEGW vector (GFP expressing; Lois et al., 2002; Holland et al., 2016) downstream of an H1 promotor and its effectiveness was tested in rat hippocampal neurons. Human mTOR cDNA mammalian expression construct (peYFP-C1 mTOR) was from the Addgene Plasmid Repository (#73384, Cambridge, MA, USA; Jain et al., 2014). Human ATP6V1A1 cDNA was from the DNASU Plasmid Repository and was subcloned into the FEWmyc vector. cDNAs for mouse RagC, human MIOS, and mouse FLCN were from Transomic Technologies (Huntsville, AL, USA) and cDNA for mouse RagD was from DNASU and were all subcloned into FEWmyc (Lois et al., 2002; Holland et al., 2016). Mouse NPRL2 cDNA was from Origene (Rockville, MD, USA) and was subcloned into the FEW vector with a C-terminal HA tag. Mouse RagA and RagB cDNA mammalian expression constructs (pCMV6-entry) were from OriGene and were tagged C-terminally with Myc and DDK tags.

### Transfections

HEK293T cells were transfected using a calcium phosphate transfection protocol as previously described (Thomas et al., 2005). Hippocampal neurons on coverslips were transfected at DIV9 with Lipofectamine 2000 (Thermo Fisher Scientific) as described (Thomas et al., 2012).

### Acyl Biotin Exchange Assay (ABE)

ABE was performed as previously described (Thomas et al., 2012). Briefly, HEK293T cells or neurons were lysed in lysis buffer (50 mM HEPES pH 7.0, 2% SDS, 1 mM EDTA plus PIC) with 20 mM methyl-methane thiosulfonate (MMTS, Thermo Fisher Scientific) or N-ethylmaleimide (NEM, Thermo Fisher Scientific) to block free cysteines. Protein was precipitated and excess MMTS or NEM removed by acetone precipitation. Protein pellets were dissolved in 4% SDS buffer and then split in two and incubated with 1 mM Biotin-HPDP (Soltec Ventures, Beverly, MA, USA) plus either pH 7.4 0.7 M hydroxylamine (NH_2_OH, HAM, Thermo Fisher Scientific) to cleave thioester bonds and remove the palmitate and label the newly revealed cysteines with biotin or with pH 7.4 50 mM Tris (buffer control). Protein was then precipitated with acetone to remove excess HAM and biotin-HPDP, re-dissolved in lysis buffer without MMTS or NEM, diluted to 0.1% (w/v) SDS with 50 mM HEPES pH 7.0 with 150 mM NaCl, and biotinylated proteins affinity-purified using neutravidin-conjugated beads (Thermo Fisher Scientific). Purified palmitoyl-proteins were then eluted using 1% (v/v) β-mercaptoethanol (Millipore-Sigma) to cleave HPDP and then denatured using SDS SB and Western blotted as described above.

### Lentiviral Infection and shRNA Knockdown

VSV-G pseudotyped lentivirus was generated in HEK293T cells as previously described (Thomas et al., 2012), by transfecting FEGW vector (with or without *Lamtor1* shRNA) plus VSV-G, pMDLg, and RSV-Rev helper plasmids. Lentivirus was collected from the HEK293T cell media 48 and 72 h post-transfection and concentrated by ultracentrifugation. Viral particles were resuspended in Neurobasal media and hippocampal neurons were transduced at DIV9. Neurons were then stimulated with BDNF as described above at DIV16.

### Immunostaining and Live Cell Imaging

Transfected neurons on coverslips were fixed in 4% (w/v) paraformaldehyde (Electron Microscopy Sciences, Hatfield, PA, USA) and 4% (w/v) sucrose in PBS for 10 min, permeabilized for 10 min using 0.25% (w/v) Triton X-100 in PBS, and blocked in 10% normal goat serum (NGS, Southern Biotech) in PBS for 1 h. Coverslips were incubated overnight at 4 degrees with primary antibodies in blocking buffer and 1 h at room temperature the following day with Alexa-Fluor conjugated secondary antibodies before mounting on glass slides with FluorSave Reagent (Millipore-Sigma, #345789). For EEA1 immunostaining, neurons were fixed in 4% (w/v) formaldehyde in PBS for 15 min, permeabilized and blocked for 1 h using 0.3% (w/v) Triton X-100 in PBS with 5% NGS. Coverslips were then incubated overnight with primary antibodies in 0.3% (w/v) Triton X-100 in PBS with 1% BSA prior to incubation with secondary antibodies and mounted as above. For Proteostat staining (#ENZ-51035-0025, Enzo Life Sciences, Inc., Farmingdale, NY, USA), neurons were fixed in 4% (w/v) formaldehyde for 30 min and stained according to the manufacturer’s protocol followed by immunostaining according to the first protocol described above. Positive control cells were treated overnight with 10 μM MG132 provided in the Proteostat kit. For LysoTracker live imaging, neurons were treated with 25 nM LysoTracker Deep Red (#L12492, Thermo Fisher Scientific) for 30 min followed by a 30 min washout in conditioned media, after which they were imaged live, as described for immunostained neurons below, in ACSF.

#### Image Acquisition

Transfected immunostained neurons and live neurons in ACSF were imaged using a Nikon C2 inverted confocal microscope with a 60× oil immersion objective (1.4 numerical aperature, plan-Apo). Z-stack images were taken with 0.15 μm spacing at 1,024 × 1,024 resolution and parameters were kept constant between different conditions. Maximum intensity projections were generated using ImageJ Fiji software for all images except those using KDEL-CFP for which two individual stacks are shown (Schindelin et al., 2012; Schneider et al., 2012).

## Results

### Multiple mTORC1 Signaling Proteins Are Putative Palmitoyl-Proteins

mTOR is rapidly recruited to lysosomal membranes during its activation, while other mTOR pathway proteins are continuously present on membranes (Sancak et al., 2010; Efeyan et al., 2012; Betz and Hall, 2013; Averous et al., 2014). To address whether palmitoylation might account for either transient or long-lived membrane localization of mTOR pathway proteins, we examined two recent curations of palmitoyl-proteomic studies (Blanc et al., 2015; Sanders et al., 2015). This search revealed that the mTOR pathway contains multiple putative palmitoyl-proteins, including subunits of GATOR1 and 2, Ragulator, TSC, and mTORC1 complexes, as well as subunits of the vacuolar ATPase (V-ATPase) and also other regulatory proteins (Figure 1, Table 1). In addition, NPRL2, a component of the GATOR1 regulator complex (Bar-Peled et al., 2013), and Folliculin (FLCN), a Rag GTPase (Petit et al., 2013), are strongly bioinformatically predicted to be palmitoylated (Ren et al., 2008; Blanc et al., 2015). Importantly, only LAMTOR1 and V-ATPase subunit a1 of the V0 sector (ATP6V0A1) have been shown to be palmitoylated in low-throughput validation studies (Martin and Cravatt, 2009; Bagh et al., 2017). Thus, palmitoylation of one or a number of these mTOR pathway proteins could provide a mechanism to recruit mTORC1 signaling complexes to lysosomal membranes, hence facilitating mTOR activation.

### Activation of mTORC1 by Amino Acids Is Palmitoylation-Dependent in Heterologous Cells

**Figure 2:**
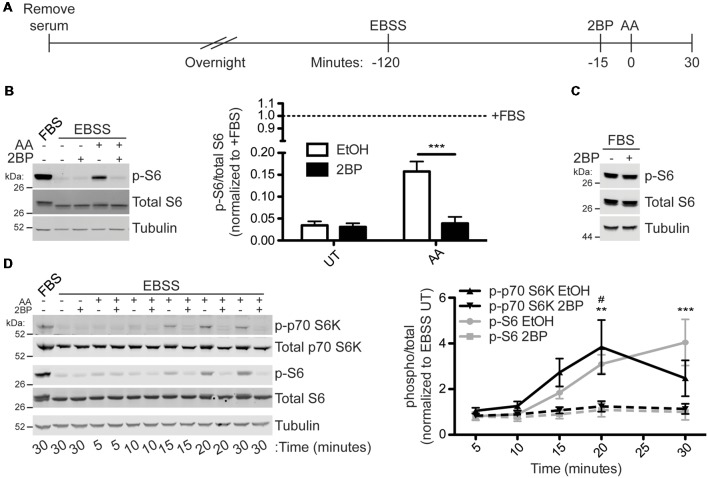
Amino acid (AA) activation of mTOR Complex 1 (mTORC1) in HEK293T cells is palmitoylation dependent. **(A)** Scheme of the AA stimulation paradigm. HEK293T cells were incubated in serum-free media 16–20 h (overnight) prior to being placed in Earle’s Balanced Salt Solution (EBSS). One hour and 45 min later cells were treated with EtOH or 20 μM 2-bromopalmitate (2BP) to inhibit palmitoylation. Fifteen minutes later cells were stimulated with 2× MEM amino acids (AA) for 30 min or left unstimulated. **(B)** Lysates from cells stimulated as in **A** were blotted to detect phospho-S6-Ser240/244 (p-S6, top, sites directly phosphorylated by p70 S6K), S6 (middle), and tubulin (bottom) levels. Quantifications are shown to the right of the Western blot images and are normalized to the serum-containing (+FBS) condition [two-way ANOVA: AA *p* = 0.0034 (*F*_(1)_ = 16.89), 2BP *p* = 0.0023 (*F*_(1)_ = 19.63), interaction *p* = 0.0047 (*F*_(1)_ = 14.99); *N* = 3; Bonferroni *post hoc* test ***p < 0.001]. **(C)** HEK293T cells in regular growth media with serum were treated with 20 μM 2BP for 45 min and lysates were blotted for p-S6 (top), S6 total (middle), and tubulin (bottom) levels. **(D)** A time course of amino acid activation of mTORC1 in HEK293T cells was performed using the same stimulation paradigm as in **A** but cells were harvested at the indicated times post stimulation with AA. Lysates were blotted to detect phospho-p70 S6 kinase-T389 (p-p70 S6K, top, a site directly phosphorylated by mTOR), p70 S6K total (second from top), p-S6 (middle), S6 total (second from bottom), and tubulin (bottom) levels. The right panel histogram shows quantified data from multiple experiments normalized to the EBSS untreated (UT) condition [two-way ANOVA for p-p70 S6K: treatment *p* = 0.001 (*F*_(1)_ = 14.69), time *p* = 0.032 (*F*_(4)_ = 3.29), interaction p = ns (*F*_(4)_ = 1.84); two-way ANOVA for p-S6: treatment *p* < 0.001 (*F*_(1)_ = 36.30), time *p* < 0.001 (*F*_(4)_ = 10.81), interaction *p* = 0.0008 (*F*_(4)_ = 7.54); *N* = 3; Bonferroni *post hoc* test ^#^*p* < 0.05 between EtOH and 2BP for p-p70 S6K and **p < 0.01, **p < 0.001 between EtOH and 2BP for p-S6].

To test the hypothesis that activation of mTORC1 is palmitoylation-dependent, HEK293T cells were subjected to a starvation paradigm prior to stimulation with amino acid (AA) for 30 min (Figure 2A). Consistent, with prior reports (Blommaart et al., 1995; Hara et al., 1998; Manifava et al., 2016), AA stimulation robustly activated mTORC1, as assessed by phosphorylation of ribosomal S6 protein (p-S6) at Ser240/244. These sites are directly phosphorylated by the mTORC1 effector p70 S6 kinase and are widely used as a downstream readout of mTOR activity (Krieg et al., 1988; Roux et al., 2007; Sun et al., 2018). Strikingly, acute pharmacological inhibition of palmitoylation with the broad spectrum palmitoylation inhibitor 2BP (Jennings et al., 2009) completely prevented AA-induced mTORC1 activation (Figure 2B). S6 phosphorylation was also highly palmitoylation-dependent in HeLa cells subjected to the same starvation/AA stimulation paradigm (Supplementary Figure S1). Importantly, acute 2BP treatment did not alter basal p-S6 levels in the absence of starvation and AA stimulation (Figure 2C). To determine the kinetics of mTORC1 activation following amino acid treatment, HEK293T cells were harvested 5, 10, 15, 20, and 30 min following AA stimulation. Phosphorylation of p70 S6 kinase at T389, a site directly phosphorylated by mTOR, increased rapidly and peaked at 20 min, whereas p-S6 levels continued to rise up to the 30-min time point (Figure 2D). 2BP prevented AA-induced phosphorylation of these two downstream targets of mTORC1 at all time points (Figure 2D). These data suggest that AA-induced activation of mTORC1 in non-neuronal cells is palmitoylation-dependent.

### Activation of mTORC1 by Neurotrophins Is Palmitoylation-Dependent in Hippocampal Neurons

**Figure 3:**
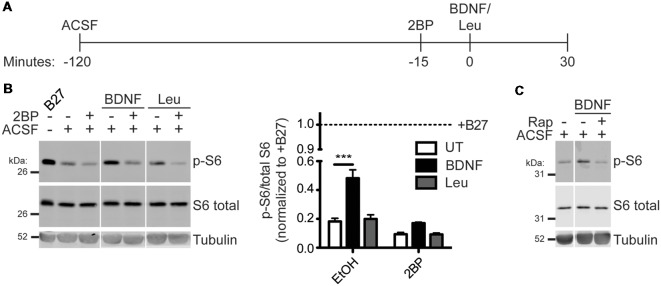
Brain-derived neurotrophic factor (BDNF) activation of mTORC1 in hippocampal neurons is palmitoylation-dependent. **(A)** Scheme of the BDNF stimulation paradigm. DIV14–16 primary rat hippocampal neurons were placed in artificial cerebrospinal fluid (ACSF) for 2 h before stimulation with 100 ng/mL BDNF or 5 mM Leu. Fifteen minutes prior to stimulation, cells were treated with ethanol (EtOH) or 50 μM 2BP to inhibit palmitoylation. Neurons were stimulated for 30 min with BDNF or Leu. **(B)** Lysates were blotted to detect p-S6 (top), S6 total (middle), and tubulin (bottom) levels. Right panel histogram shows quantified data from multiple experiments, normalized to the regular growth media with B27 (+B27) condition [two-way ANOVA: 2BP *p* < 0.0001 (*F*_(2)_ = 28.41), treatment *p* < 0.0001 (*F*_(1)_ = 59.31), interaction *p* = 0.0017 (*F*_(2)_ = 9.52); *N* = 3–5; Bonferroni *post hoc* test ***p < 0.001]. Panels are composites of the same Western blot image. **(C)** Neurons were stimulated as in **B** with BDNF, but 15 min prior to stimulation cells were treated with EtOH or 100 nM rapamycin (Rap) to inhibit mTOR.

To determine if activation of mTORC1 is also palmitoylation-dependent in neurons, hippocampal neurons were acutely incubated in amino-acid free medium artificial cerebrospinal fluid (ACSF) and then stimulated with BDNF or Leucine (Leu) in conjunction with acute 2BP treatment (Figure 3A). BDNF stimulation robustly activated mTORC1 in hippocampal neurons, as measured by p-S6 levels, and this p-S6 increase was prevented by 2BP (Figure 3B). Although Leu alone did not significantly increase p-S6, 2BP reduced basal p-S6 levels in both the presence and absence of Leu (Figure 3B). These findings suggest that palmitoylation is important for both BDNF-induced and basal mTORC1 activity in neurons, but that, in contrast to non-neuronal cells and cortical neurons (Blommaart et al., 1995; Hara et al., 1998; Ishizuka et al., 2008) Leucine does not significantly activate mTORC1 in hippocampal neurons. Importantly, BDNF-induced mTORC1 activation was blocked by acute application of rapamycin, a direct mTOR inhibitor, consistent with established models that S6 phosphorylation is controlled “downstream” of mTOR (Figure 3C). Thus, activation of mTORC1 in neurons to a physiologically relevant stimulus, BDNF, is also palmitoylation-dependent.

### A Screen of Putatively Palmitoylated mTOR Pathway Proteins Reveals That LAMTOR1 and mTOR Are Palmitoylated

**Figure 4:**
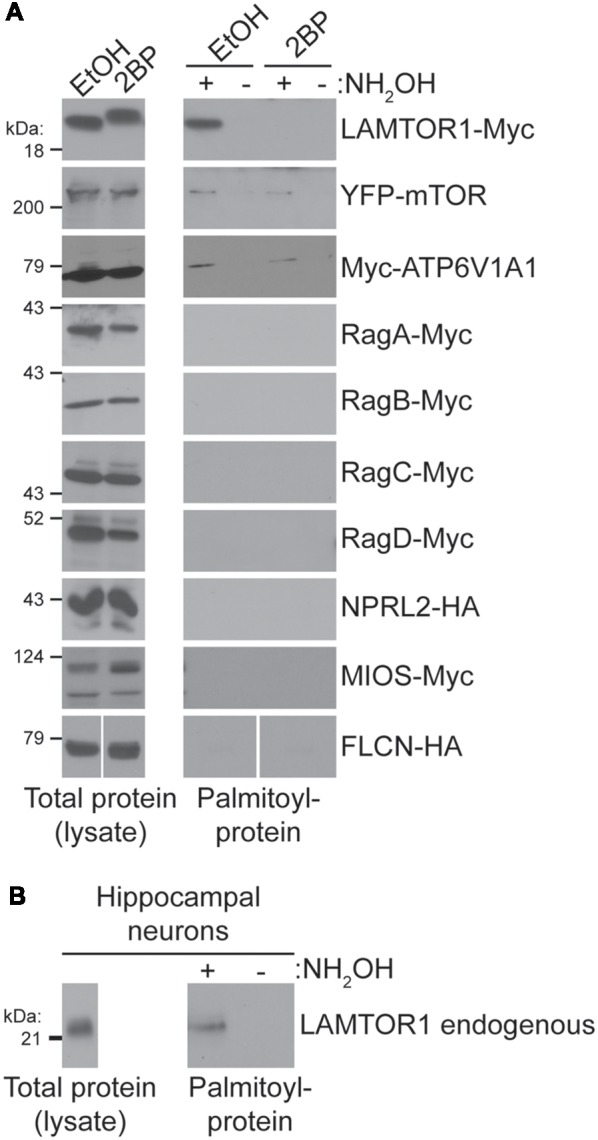
**(A)** Candidate-based screen of putative palmitoylated proteins of the mTOR signaling pathway. HEK293T cells were transfected with the indicated constructs for 8 or 24 (mTOR only) hours and were treated with 100 μM 2BP or EtOH at 4 h post-transfection. Palmitoyl-proteins [isolated by acyl biotin exchange (ABE); right panels] and total protein levels (in parent lysates; left panels) were assessed by western blotting with antibodies against the indicated tags. FLCN-HA panels are composites of the same Western blot image. **(B)** LAMTOR1 is palmitoylated in hippocampal neurons. Total LAMTOR1 (left panel) and palmitoyl-LAMTOR1 level (right panel) from DIV16 hippocampal neurons detected by western blotting.

To identify the palmitoyl-protein(s) that might be responsible for palmitoylation-dependent mTORC1 activation we took a candidate-based approach. In particular, we assessed palmitoylation of epitope-tagged versions of the following proteins: LAMTOR1, mTOR, ATP6V1A1, RagA-D, NPRL2, MIOS, and FLCN. Palmitoyl-proteins were isolated from transiently transfected HEK293T cells using ABE, a non-radioactive method to isolate palmitoyl-proteins from cell lysates. Myc-tagged LAMTOR1 (LAMTOR1-Myc), a subunit of the “Ragulator” required for lysosomal membrane localization of this complex (Sancak et al., 2010), was robustly detected in ABE fractions from cells treated with vehicle (EtOH), but was absent from ABE fractions from cells treated with 2BP or in negative control fractions lacking the essential ABE component hydroxylamine (NH_2_OH; Figure 4A). Palmitoylation of LAMTOR1 (also known as p18 or c11orf59) was previously validated in a low-throughput experiment (Martin and Cravatt, 2009). Consistent with these findings, we also observed robust palmitoylation of endogenous LAMTOR1 in ABE fractions from hippocampal neurons (Figure 4B). YFP-mTOR and Myc-ATP6V1A1 were also weakly palmitoylated in our HEK293T cell ABE assays, but no other protein tested was palmitoylated (Figure 4A). Thus, although many mTOR pathway proteins localize to lysosomal membranes and have been identified in palmitoyl-proteomic studies, only a specific subset of these proteins is palmitoylated under the conditions that we examined.

### Palmitoylation of LAMTOR1 Rapidly Increases and Palmitoylation of mTOR Decreases Following Amino Acid Stimulation in HEK293T Cells

**Figure 5:**
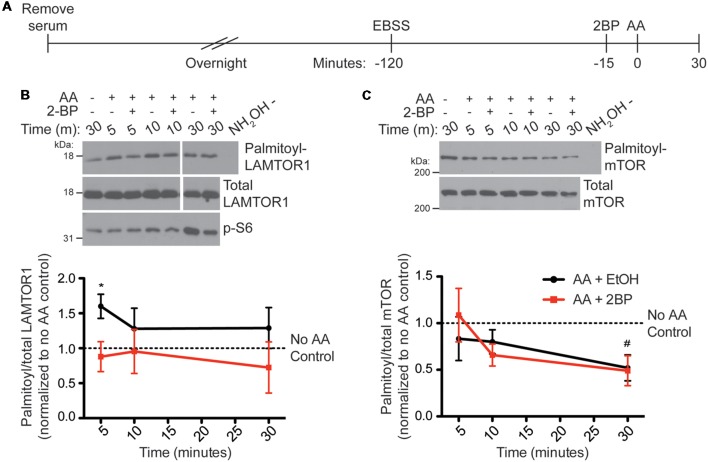
LAMTOR1 palmitoylation is rapidly increased and mTOR palmitoylation is decreased by amino acid stimulation (of mTORC1) in HEK293T cells. **(A)** Scheme of the AA stimulation paradigm. HEK293T cells were incubated in serum-free media 16–20 h (overnight) prior to being placed in EBSS. One hour and 45 min later cells were treated with ethanol (EtOH) or 20 μM 2BP to inhibit palmitoylation. Cells were stimulated 15 min later with 2× MEM amino acids (AA) and harvested at 5, 10, and 30 min post-stimulation. Control cells were lysed 30 min after sham stimulation with buffer alone. Palmitoyl-proteins were isolated by ABE and samples were blotted for LAMTOR1 **(B)** and mTOR **(C)** in the ABE fraction (top) and total lysate input fraction (middle). Lysates were also blotted for pS6 (bottom panels). Panels are composites of the same Western blot image. Lower histograms show quantified data from *N* = 4 experiments [*Student’s *t*-test with Welch’s correction vs. unstimulated condition (no AA control, first lane of western blot) for LAMTOR1, *p* = 0.040; ^#^Student’s *t*-test with Welch’s correction vs. unstimulated condition (no AA control, first lane of western blot) for mTOR, *p* = 0.041].

To determine if palmitoylation of LAMTOR1 or mTOR change in response to AA stimulation of HEK293T cells, which may help explain the palmitoylation-dependence of mTORC1 activation, we used ABE to isolate palmitoyl-proteins from HEK293T cells following AA stimulation (Figure 5A). Interestingly, LAMTOR1 palmitoylation rapidly increased more than 1.5-fold between 5 and 10 min post-stimulation and this increase was blocked by 2BP (Figure 5B). This finding suggests that LAMTOR1 palmitoylation is dynamically regulated during mTOR activation. Under the same conditions, we observed a slower decrease in palmitoyl-mTOR levels in ABE fractions (Figure 5C), suggesting that prolonged mTOR activation correlates with decreased mTOR palmitoylation.

### Palmitoylation Controls LAMTOR1 Subcellular Localization in Neurons

**Figure 6:**
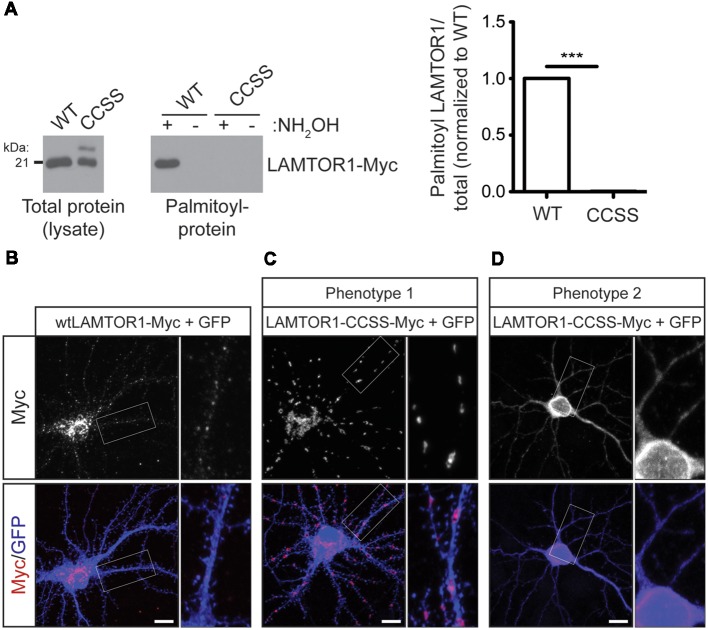
Palmitoylation is required for LAMTOR1 localization in hippocampal neurons. **(A)** Mutation of cysteines 3 and 4 in Myc-tagged human LAMTOR1 (LAMTOR1-CCSS-Myc) abolishes its palmitoylation. Total expression (left panel) and palmitoyl-levels (right panel) of WT or CCSS LAMTOR1-Myc isolated from transfected HEK293T cells. Right histogram shows quantified palmitoyl/total LAMTOR1 levels (Student’s *t*-test with Welch’s correction, *** < 0.001, *N* = 3). **(B)** Hippocampal neurons were transfected to express wtLAMTOR1-Myc and morphology marker GFP. *First* *column*: images of Myc and merged images of Myc/GFP signals. *Second column*: magnified views of the boxed area of first column images. **(C,D)** As in **A** except that neurons were transfected with LAMTOR1-CCSS-GFP. **(C)** Phenotype 1 of LAMTOR1-CCSS-Myc, observed in approx. 85% of neurons and **(D)** phenotype 2 of LAMTOR1-CCSS-Myc, observed in approx. 15% of neurons. Scale bar indicates 10 μm.

We next sought to define the functional role of LAMTOR1 palmitoylation in mTOR signaling by generating a LAMTOR1 mutant in which two cysteine residues, C3 and C4, were mutated to non-palmitoylatable serine (LAMTOR1-CCSS). Although C3 and C4 are the only cysteines present in LAMTOR1 and are required to anchor the Ragulator to lysosomes (Nada et al., 2009), whether these sites are directly palmitoylated has not been previously addressed. In contrast to wild type LAMTOR1, LAMTOR1-CCSS was absent from ABE fractions, confirming that LAMTOR1-C3 and -C4 are *bona fide* palmitoylation sites (Figure 6A).

We then used the LAMTOR1-CCSS mutant to assess possible roles of LAMTOR1 palmitoylation in neurons. In addition to the dual palmitoylation sites at C3 and C4, LAMTOR1 also contains a glycine residue at position 2 that is highly predicted to become myristoylated following cleavage of the N-terminal methionine (Nada et al., 2009; Martin and Hayden, 2015). Myristoylation allows proteins to sample membranes but does not provide stable attachment without a secondary membrane binding signal, such as palmitoylation (Peitzsch and McLaughlin, 1993; Martin et al., 2011). While wtLAMTOR1-myc localized to small puncta that resemble lysosomes (Figure 6B), LAMTOR1-CCSS-myc localized to larger, tubular structures in the majority (approximately 85%) of transfected neurons (Figure 6C; phenotype 1). In approximately 15% of transfected neurons, LAMTOR1-CCSS-Myc was more diffusely localized (Figure 6D; phenotype 2).

**Figure 7:**
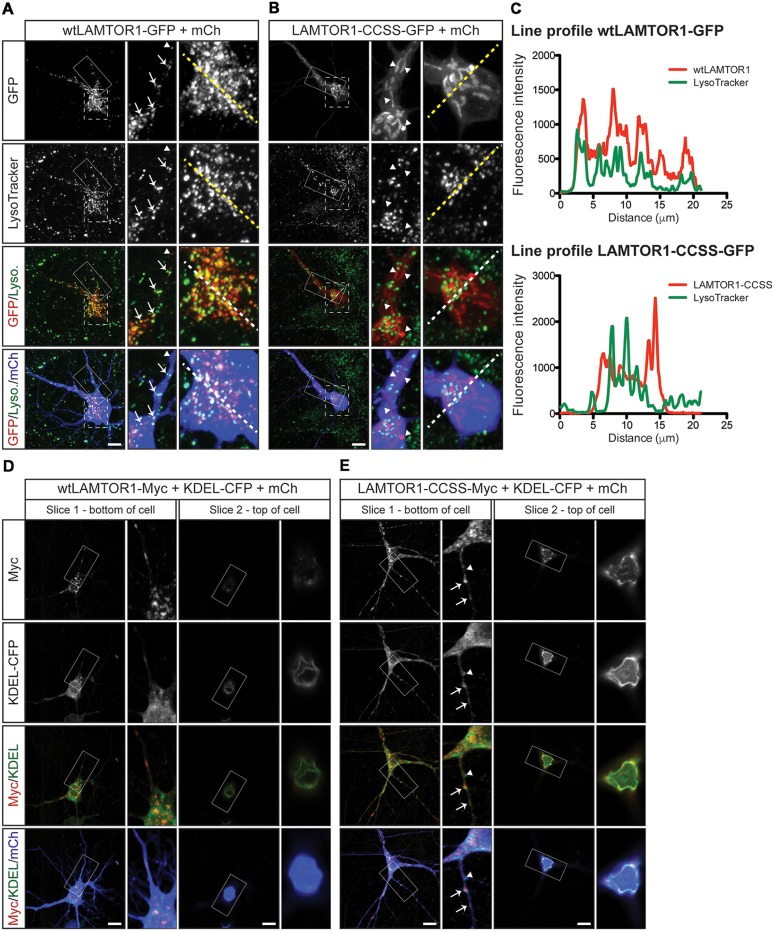
Palmitoylated wtLAMTOR1 localizes to lysosomes whereas LAMTOR1-CCSS partially localizes to endoplasmic reticulum (ER) membranes. **(A)** Hippocampal neurons were transfected to express wtLAMTOR1-GFP and morphology marker mCherry (mCh) prior to incubation with LysoTracker dye and subsequent live imaging. *First* *column*: images of GFP and LysoTracker signals and merged images of GFP/LysoTracker (Lyso.) and GFP/Lyso./mCh signals. *Second column*: magnified views of the solid boxed area of first column images. Arrows indicate LAMTOR1-GFP positive vesicles that are also LysoTracker positive Arrowheads indicate LAMTOR1-GFP puncta that are LysoTracker negative. *Third column*: magnified views of the dotted boxed area of first column images. **(B)** As in **A** except that neurons were transfected with LAMTOR1-CCSS-GFP. **(C)** Graphs of the fluorescent intensity profiles of wt (top) and CCSS (bottom) LAMTOR-GFP and LysoTracker along the dotted lines indicated in the third columns of **A,B**, respectively. wt-LAMTOR1 signal closely matches the profile of LysoTracker signal while LAMTOR1-CCSS signal does not. **(D)** *First column*: single slice confocal images from a region close to the bottom of the cell of a hippocampal neuron transfected to express wtLAMTOR1-Myc, the ER marker KDEL-CFP (detected using anti-GFP antibody), and mCh, and merged images of Myc/KDEL-CFP (KDEL) and Myc/KDEL/mCh signals. *Second column*: magnified views of the boxed area of first column images. *Third and fourth columns*: as the first and second columns, respectively, except images show a single confocal slice from a region close to the top of the cell. **(E)** As in **D** except that neurons were transfected to express LAMTOR1-CCSS-Myc. Arrows indicate LAMTOR1-CCSS-Myc tubules that are positive for KDEL-CFP, arrowhead indicates LAMTOR1-CCSS-Myc KDEL-CFP negative tubule. Scale bar indicates 10 μm.

To characterize the wtLAMTOR1 puncta and LAMTOR1-CCSS tubular structures, we assessed their colocalization with subcellular markers. We first expressed GFP tagged wt and CCSS LAMTOR1 in neurons and acquired images of live cells using LysoTracker, a well-characterized lysosomal marker (Chazotte, 2011). Consistent with prior reports (Sancak et al., 2010; Sun et al., 2018) the majority of wtLAMTOR1-GFP puncta were LysoTracker positive (Figure 7A; arrows). In contrast, the LAMTOR1-CCSS-GFP tubular structures were predominantly LysoTracker negative (Figure 7B; arrowheads). Individual line profiles confirmed the clear overlap of wtLAMTOR1, but not LAMTOR1-CCSS, with LysoTracker (Figure 7C).

To identify the tubular structures of LAMTOR1-CCSS, we examined LAMTOR1 co-localization with additional markers. Although, wtLAMTOR1 showed little co-localization with the ER marker KDEL-CFP (Thomas et al., 2012; Figure 7D), LAMTOR1-CCSS partially co-localized with KDEL-CFP, particularly towards the top of the cell soma (Figure 7E) and, to a lesser extent, within dendrites (Figure 7E; arrows in inset). These findings suggested that LAMTOR1-CCSS localizes, in part, to the ER. However, an alternative possibility is that the LAMTOR1-CCSS tubule structures could represent aggregates, potentially including other proteins. To address this possibility, we stained wt and CCSS LAMTOR1 expressing neurons with the aggresome dye Proteostat (Shen et al., 2010). We detected no positive Proteostat signal that co-localized with wt or CCSS LAMTOR1 (Supplementary Figures S2A,B, respectively), despite strong positive signal in cells treated with the proteosomal inhibitor MG132 (Supplementary Figure S2C; right panels). These results suggest that the CCSS tubule structures are not aggregates. We also examined the colocalization of wt and CCSS LAMTOR1 with markers for early endosomes (EEA1) and Golgi (GM130). Both wt and CCSS LAMTOR1-Myc co-localized with a small number of early endosomes (Supplementary Figures S2D,E, respectively; arrows in inset) but neither form of LAMTOR1 colocalized with GM130 (Supplementary Figures S2F,G). Taken together, these results suggest that palmitoylation predominantly targets LAMTOR1 to lysosomes in neurons. In addition, these findings suggest that the “myristoyl-only” LAMTOR1-CCSS mutant still localizes to membranes in neurons, in contrast to the diffuse localization of this mutant reported in non-neuronal cells (Nada et al., 2009). However, LAMTOR1 palmitoyl-site mutation results in mistargeting, in part to the ER.

### LAMTOR1 Is not Required for Neurotrophin-Induced Activation of mTORC1 in Hippocampal Neurons

**Figure 8:**
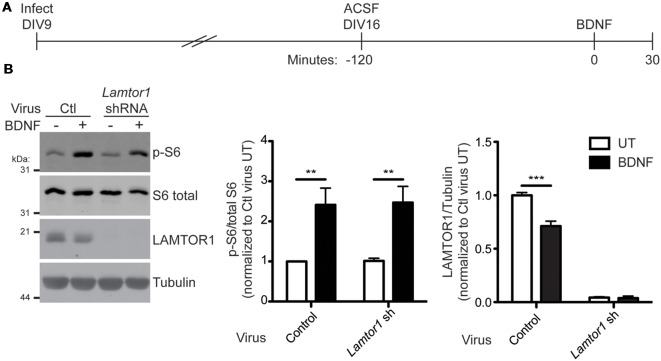
LAMTOR1 is not required for BDNF activation of mTORC1 in hippocampal neurons. **(A)** Scheme of the viral infection and BDNF stimulation paradigm. Hippocampal neurons were infected with control (Ctl) or *Lamtor1* shRNA lentivirus at 9 days *in vitro* (DIV). On DIV16, cells were placed in ACSF for 2 h before stimulation with 100 ng/ml BDNF for 30 min. **(B)** Lysates from samples treated as in **A** were blotted to detect p-S6 (top), S6 total (second from top), LAMTOR1 (second from bottom), and tubulin (bottom) levels. Quantifications of pS6 relative to total S6 [two-way ANOVA: BDNF treatment *p* = 0.0002 (*F*_(1)_ = 24.07), virus *p* = 0.91 (*F*_(1)_ = 0.013), interaction *p* = 0.94 (*F*_(1)_ = 0.0052); *N* = 5; Bonferroni *post hoc* test **p < 0.01) and of LAMTOR1 relative to tubulin [two-way ANOVA: BDNF treatment *p* = 0.0009 (*F*_(1)_ = 26.60), virus *p* < 0.0001 (*F*_(1)_ = 822.6), interaction *p* = 0.001 (*F*_(1)_ = 25.04); *N* = 3; Bonferroni *post hoc* test ***p < 0.001] are shown to the right of the Western blot images and are normalized to the control untreated (UT) condition.

mTORC1 activation by multiple stimuli requires LAMTOR1 (Kimura et al., 2016; Hosokawa et al., 2017) and loss of LAMTOR1 is associated with decreased basal mTORC1 activity in hippocampal neurons (Sun et al., 2018). To determine if LAMTOR1 is required for BDNF-induced activation of mTORC1 in hippocampal neurons, we lentivirally delivered *Lamtor1* shRNA into neurons and subsequently stimulated neurons with BDNF (Figure 8A). Interestingly, BDNF-induced mTORC1 activation (as measured by S6 phosphorylation at specific p70 S6K phosphorylation sites) occurred normally in *Lamtor1*-deficient neurons, despite the reduction of LAMTOR1 protein levels by >95% (Figure 8B). These findings suggest, surprisingly, that LAMTOR1 (and hence its palmitoylation) is not required for BDNF-induced mTOR activation. One possible explanation for this discrepancy is that LAMTOR2 or LAMTOR3 are palmitoylated in LAMTOR1 “knockdown” neurons and hence compensate for the loss of LAMTOR1. To address this possibility, we isolated palmitoyl-proteins from control or *Lamtor1* shRNA infected hippocampal neurons. We robustly detected palmitoylation of LAMTOR1 in control-infected neurons and GAP43, a well known neuronal palmitoyl-protein, in both control and *Lamtor1*-deficient neurons. However, we were unable to detect palmitoylation of LAMTOR2 or LAMTOR3 in control or *Lamtor1* shRNA infected neurons (Supplementary Figure S3). These results suggest that palmitoylation of LAMTOR2 and/or LAMTOR3 is not a compensatory mechanism for the loss of LAMTOR1.

## Discussion

mTORC1 signaling plays a key role in the regulation of protein synthesis, transcription, cytoskeletal regulation and autophagy, and is also essential for normal neuronal development and function (Sancak et al., 2008, 2010; Efeyan et al., 2012; Betz and Hall, 2013; Averous et al., 2014). A key step in mTORC1 activation by multiple stimuli is the translocation of mTOR itself to lysosomes (Sancak et al., 2008). Many other proteins involved in mTORC1 signaling are constitutively present on lysosomes (Sancak et al., 2010; Menon et al., 2014). The mechanisms underlying the precise constitutive and stimulus-dependent localization of mTORC1 signaling proteins are not fully understood, but our findings suggest that palmitoylation is essential for mTORC1 activation by multiple stimuli.

We recognize that this conclusion stems mainly from our experiments using 2BP, a broad inhibitor of palmitoylation with potentially multiple mechanisms of action (Davda et al., 2013). However, 2BP effectively blocks mTORC1 activation after only acute incubation, and the 2BP concentration required to block mTOR signaling is very similar to that required to block other palmitoylation-dependent events in cells (Mikic et al., 2006). These two findings increase the likelihood that our observed effects of 2BP on mTORC1 activity are due to *bona fide* inhibition of palmitoylation. This conclusion is further supported by our observation that at least one mTOR pathway protein is dynamically palmitoylated in a 2BP-dependent manner, with kinetics that precedes mTORC1 activation (Figure 5). Nonetheless, we recognize that identification of the key palmitoyl-protein(s) that is the target of 2BP will be essential to conclude that the effects of this compound on mTOR signaling are direct.

LAMTOR1 palmitoylation increases rapidly in response to amino acid stimulation in HEK293T cells (Figure 5B). This effect is one of a surprisingly limited number of examples in which palmitoylation dynamically increases in response to a stimulus (El-Husseini et al., 2002; Keith et al., 2012; Poggi et al., 2012; Akimzhanov and Boehning, 2015; Brigidi et al., 2015). LAMTOR1 might thus appear to be a strong candidate to be the key protein whose palmitoylation explains the effect of 2BP on mTORC1 signaling. However, we found that acute loss of LAMTOR1 did not affect BDNF-induced mTORC1 activation in hippocampal neurons (Figure 8B). This result was initially surprising, given that knockout of LAMTOR1 blocks mTORC1 activation by multiple stimuli (Kimura et al., 2016; Hosokawa et al., 2017). However, it is important to note that the conclusion that LAMTOR1 is required for mTORC1 activation by other stimuli stemmed from studies that employed long-term genetic loss rather than acute shRNA-mediated knockdown of *Lamtor1*. It is thus possible that other factors can compensate for the loss of LAMTOR1 over acute, but not prolonged time periods. Indeed, we tested one compensatory mechanism, namely the possibility that LAMTOR1 knockdown increases palmitoylation of LAMTOR2 and/or 3. However, we did not observe palmitoylation of these other members of the Ragulator complex, either in the presence or absence of LAMTOR1 (Supplementary Figure S3). Our study also differs because we examined the requirement for LAMTOR1 in neurons rather than the non-neuronal cells assessed by Hosokawa et al. (2017) and Kimura et al. (2016). To our knowledge, only one study has assessed the impact of LAMTOR1 loss on mTOR activity in neurons, and only modest effects were observed (Sun et al., 2018). Moreover, we found that the amino acid leucine, which potently activates mTORC1 in non-neuronal cells and cultured cortical neurons (Blommaart et al., 1995; Hara et al., 1998; Ishizuka et al., 2008), does not activate mTORC1 in hippocampal neurons. Thus, there may be differences in the LAMTOR1-dependence of mTORC1 signaling in neurons vs. non-neuronal cells and/or between different neuronal types. Consistent with this notion, growth factor-induced mTORC1 activation in non-neuronal cells requires the presence of amino acids (Sancak et al., 2008, 2010), while BDNF-induced mTORC1 activation in neurons instead requires the presence of glucose (Ishizuka et al., 2013). Nonetheless, palmitoylation is clearly required for LAMTOR1 neuronal localization (Figures 6, 7) suggesting that this modification is functionally relevant for LAMTOR1-dependent neuronal signaling.

At the subcellular level, we found that wild type (palmitoylation-competent) LAMTOR1 localizes predominantly to lysosomes (Figure 7A), consistent with prior reports (Sancak et al., 2010; Sun et al., 2018), while palmitoyl-mutant LAMTOR1 is partially detected on tubular membranes that colocalize in part with an ER marker (Figure 7E). In contrast, neither LAMTOR1 wild type nor LAMTOR1-CCSS localizes to aggresomes (Supplementary Figure S2). Under the conditions that we examined, palmitoylation therefore controls LAMTOR1 subcellular localization but is less likely to affect LAMTOR1 proteostasis or aggregation. However, we cannot exclude a role for palmitoylation in these latter processes in different cell types and/or under different conditions.

mTORC1 pathway activation triggered by amino acids correlates not only with increased LAMTOR1 palmitoylation but also a slight reduction in mTOR palmitoylation (Figure 5). A *priori*, it might appear that depalmitoylation could disinhibit mTOR, favoring its activation. However, the delayed kinetics of mTOR depalmitoylation (which only becomes significant well after the peak of phosphorylation of mTOR’s substrate p70 S6K; compare Figures 2D, 5C) reduce the likelihood of this possibility. It nonetheless appears possible that delayed mTOR depalmitoylation may represent a feedback control mechanism for this pathway, a hypothesis that could benefit from additional investigation.

Given a large number of mTORC1 signaling proteins that have been identified in palmitoyl-proteomic studies (Figure 1, Table 1), we were surprised that only a subset of these proteins was palmitoylated when tested in our candidate-based screen (Figure 4). It is, of course, possible that some of these palmitoyl-proteomic identifications may be false positives, highlighting the importance of low-throughput validation. However, there are other possible explanations for this discrepancy. In particular, it is important to note that, in contrast to other protein-lipid modifications (myristoylation and prenylation), palmitoylation is reversible and does not occur co-translationally. Importantly, we assessed palmitoylation of these proteins under one specific condition in one cell type. It is thus possible that these proteins may be palmitoylated in other cell types or under other conditions (for example at a specific developmental time and/or subcellular location), which may explain their initial assignment as palmitoyl-proteins.

While we recognize that key questions remain to be addressed, our finding that mTORC1 activation is palmitoylation-dependent supports a growing number of reports that this modification not only controls protein subcellular localization but governs activity of multiple intracellular kinase pathways (Kabouridis et al., 1997; Stoffel et al., 1998; van’t Hof and Resh, 1999; Davidson et al., 2005; Tsutsumi et al., 2008; Yang and Cynader, 2011; George et al., 2015; Holland et al., 2016). Moreover, because aberrant mTORC1 signaling is central to many disease states (Laplante and Sabatini, 2012; Costa-Mattioli and Monteggia, 2013), our findings raise the possibility that pharmacological modulation of palmitoylation could be a new therapeutic approach to reduce the impact of pathological mTOR dysregulation.

## Data Availability

The datasets generated for this study are available on request to the corresponding author.

## Author Contributions

SS and FDS designed and performed the experiments. SS prepared the figures. SS and GT conceived the project and wrote the manuscript. SS, FDS, and GT edited the manuscript.

## Conflict of Interest Statement

The authors declare that the research was conducted in the absence of any commercial or financial relationships that could be construed as a potential conflict of interest.
