# Supplementary material for: mTORC1 Signaling Is Palmitoylation-Dependent in Hippocampal Neurons and Non-neuronal Cells and Involves Dynamic Palmitoylation of LAMTOR1 and mTOR
Source: Front Cell Neurosci. 2019 Apr 2;13:115. doi: 10.3389/fncel.2019.00115 (PMC6454084; doi:10.3389/fncel.2019.00115)
Supplement: Supplementary file 1 [file Data_Sheet_1.PDF]

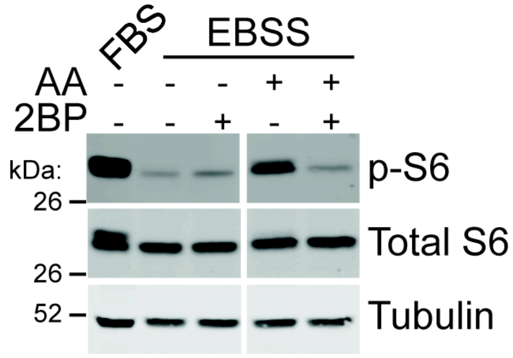

Supplemental Figure 1. mTORC1 activation by amino acids is palmitoylation dependent in HeLa cells. HeLa cells were stimulated with amino acids using the same paradigm as for HEK293T cells (Figure 2A). Cells were incubated in serum free media 16-20 hours (overnight) prior to being placed in Earle's Balanced Salt Solution (EBSS). One hour and 45 minutes later cells were treated with 20 μM 2BP or ethanol (EtOH, vehicle control) for 15 min, prior to stimulation with 2x MEM amino acids (AA) or vehicle for 30 minutes. Lysates were blotted to detect phospho-S6 (p-S6, top), S6 total (middle), and tubulin (bottom) levels. Similar results were observed in a replicate experiment. Panels are composites of the same Western blot image.

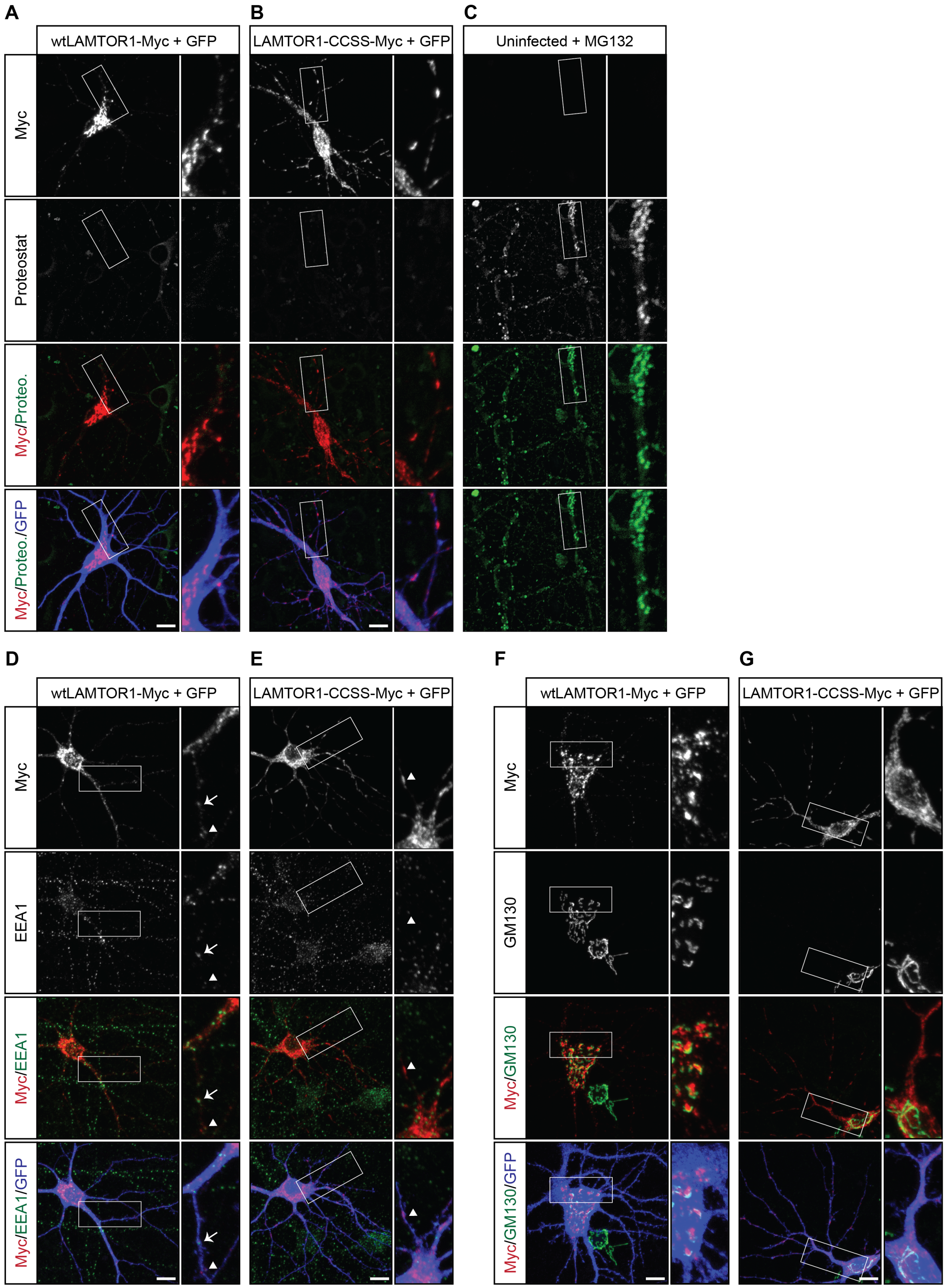

Supplemental Figure 2. wtLAMTOR1-Myc occasionally localizes to early endosomes but not to the Golgi and neither wt or CCSS LAMTOR1-Myc forms aggregates. (**A**). *First column:* images of Myc and the aggresome detecting dye Proteostat and merged images of Myc/Proteostat (Proteo.) and Myc/Proteo./GFP signals from hippocampal neurons transfected to express wtLAMTOR1-Myc and GFP and

incubated with Proteostat after fixation. *Second column:* magnified views of boxed area of first column images. (**B**) As in A except for neurons transfected to express LAMTOR1-CCSS-Myc and GFP. (**C**) As in A and B, except for untransfected hippocampal neurons treated with MG132 to block proteosomal degradation and induce protein aggregation. (**D**) *First column:* images of Myc and early endosome marker EEA1 signals and merged images of Myc/EEA1 and Myc/EEA1/GFP signals from hippocampal neurons transfected to express wtLAMTOR1-Myc and morphology marker GFP. *Second column:* magnified views of boxed area of first column images. Arrows indicate LAMTOR1-Myc positive vesicles that are also EEA1 positive, arrowheads indicate LAMTOR1-Myc puncta that are EEA1 negative. (**E**) As in D except for neurons transfected to express LAMTOR1-CCSS-Myc and GFP. (**F**) and (**G**) as is in D and E, respectively, except using the Golgi marker GM130. Scale bar indicates 10 μm.

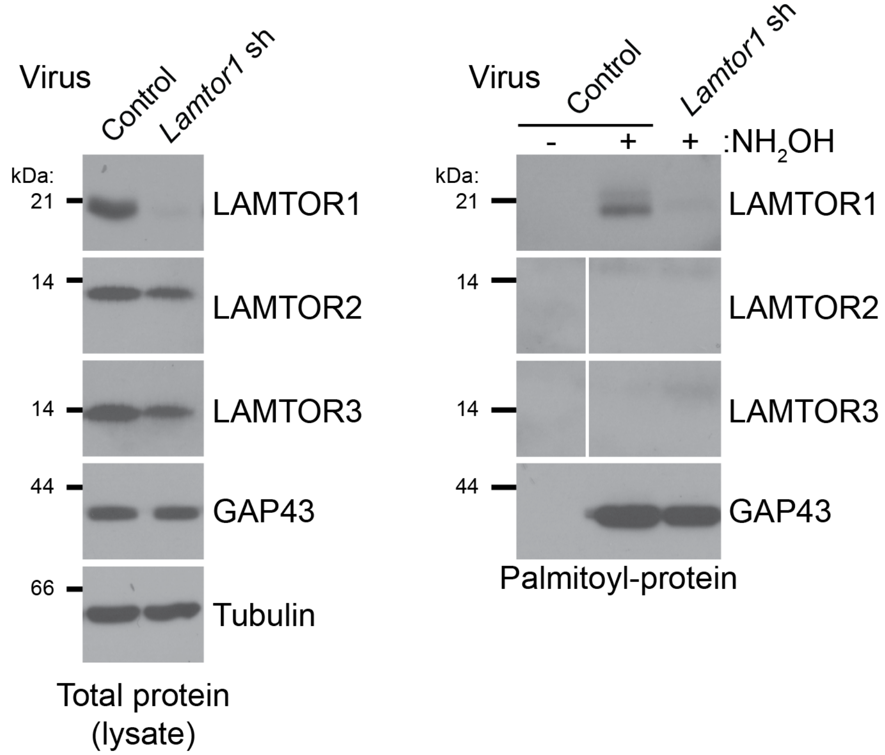

Supplemental Figure 3. Knockdown of LAMTOR1 does not trigger palmitoylation of LAMTOR2 or LAMTOR3 in hippocampal neurons. (**A**) Total expression (left panels) and palmitoyl-levels (right panels) of LAMTOR1, LAMTOR2, LAMTOR3, GAP43, and tubulin isolated from control or *Lamtor1* shRNA infected hippocampal neurons. Panels are composites of the same Western blot image.

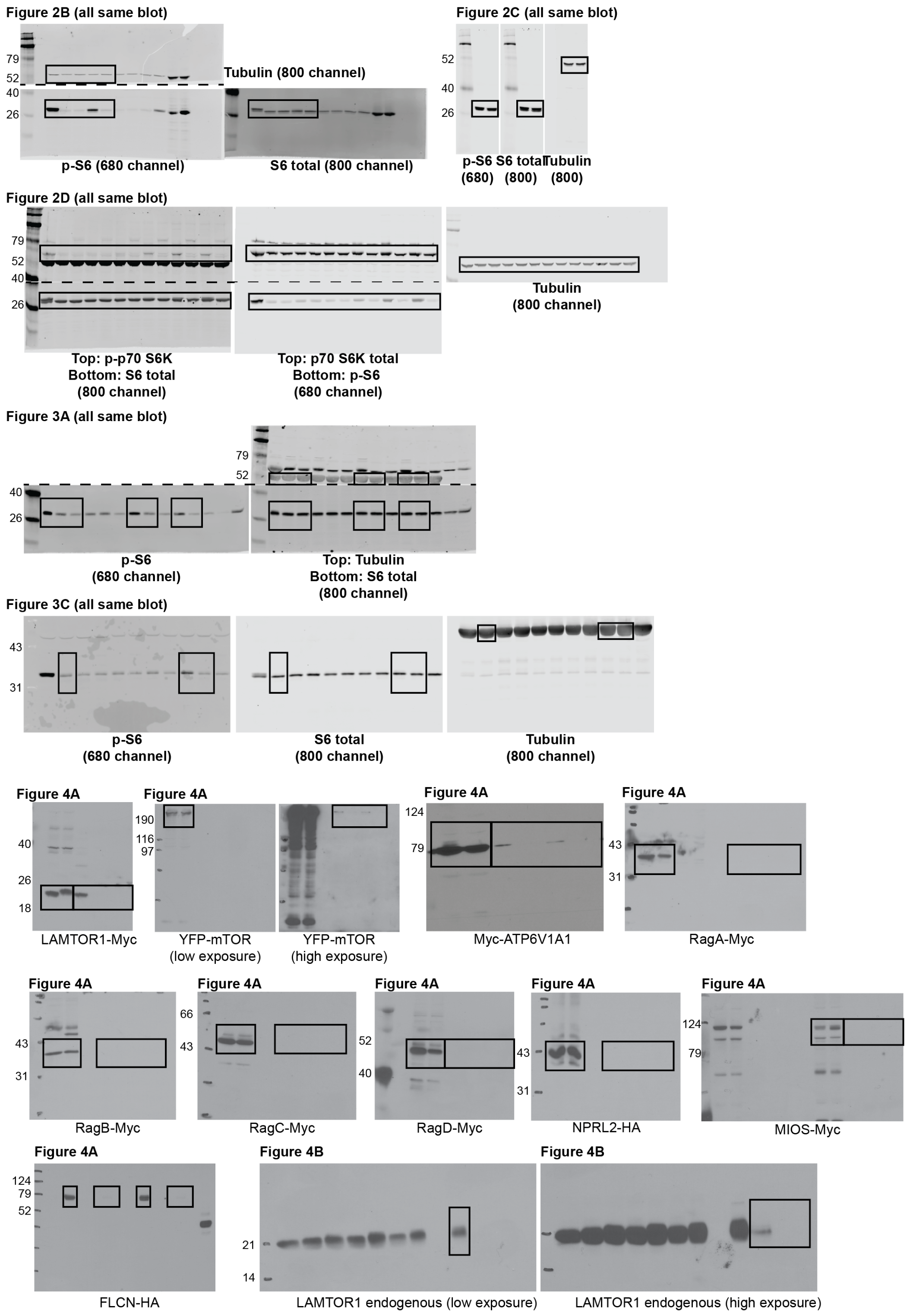

Supplemental Figure 4. Uncropped Western blot images for Figures 2, 3, and 4. Boxes indicate cropped regions and dashed lines indicate where membranes were cut.

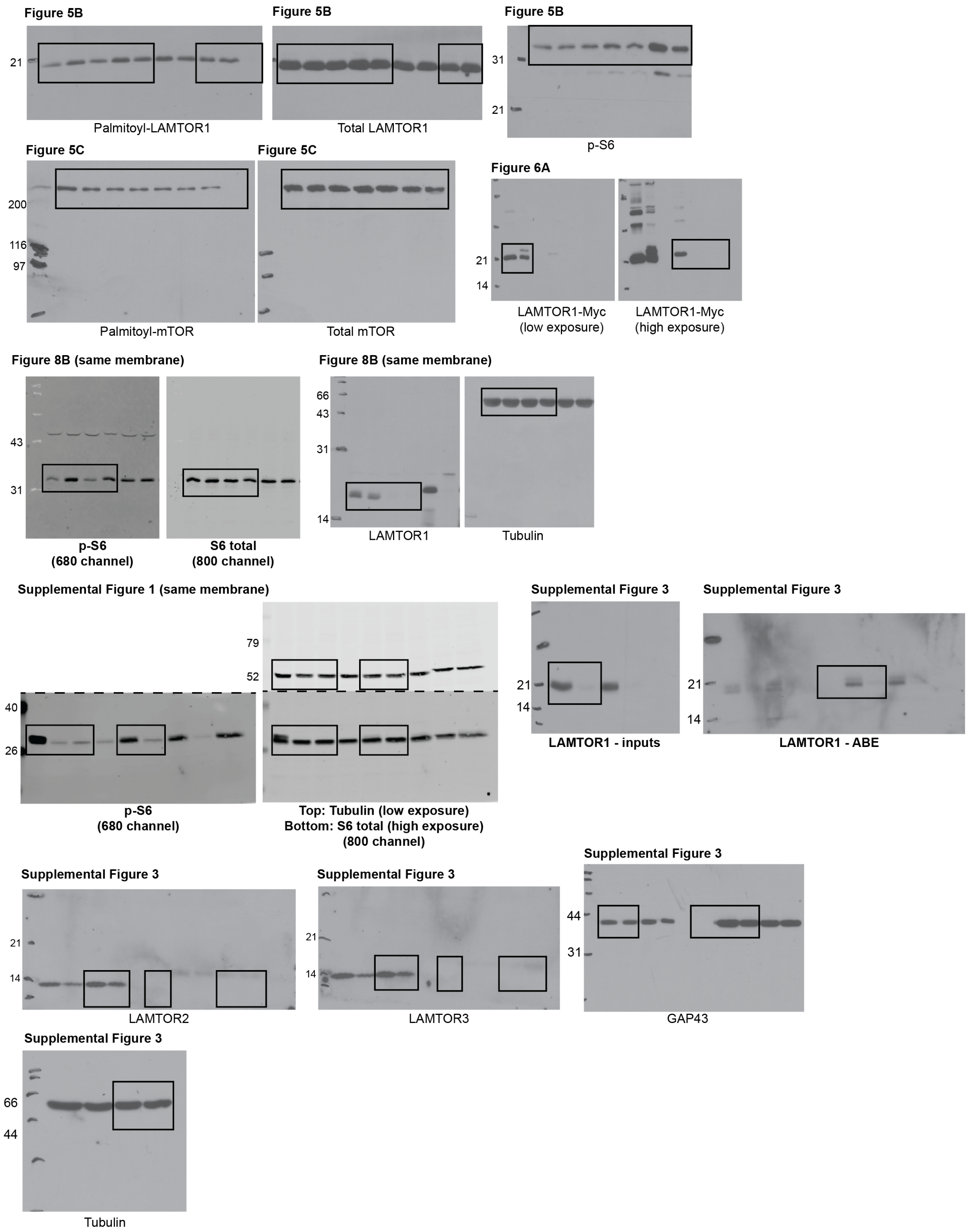

Supplemental Figure 5. Uncropped Western blot images for Figures 5, 6, 8, and Supplemental Figures 1 and 3. Boxes indicate cropped regions and dashed lines indicate where membranes were cut.
